# Child acceptability of a novel provitamin A carotenoid, iron and zinc-rich complementary food blend prepared from pumpkin and common bean in Uganda: a randomised control trial

**DOI:** 10.1186/s12887-020-02326-z

**Published:** 2020-09-01

**Authors:** Edward Buzigi, Kirthee Pillay, Muthulisi Siwela

**Affiliations:** 1 Department of Dietetics and Human Nutrition, School of Agricultural, Earth and Environmental Sciences, University of KwaZulu-Natal, Private Bag X01, Scottsville 3209, Pietermaritzburg, 3201 South Africa; 2 Health Economics and HIV/AIDS Research Division (HEARD), University of KwaZulu-Natal, Westville Campus, J Block 4th Floor, Durban, 4041 South Africa; 3 Department of Human Nutrition & Home Economics, Kyambogo University, P.O. Box 1 Kyambogo, Kampala, Uganda

**Keywords:** Child acceptability, Complementary foods, Common bean pumpkin blend, Pumpkin blend, Provitamin A carotenoids, Iron, Zinc, Vitamin A, Uganda

## Abstract

**Background:**

Ugandan children are fed homemade complementary foods (CFs) which are usually deficient in vitamin A, iron and zinc. Novel homemade CFs rich in vitamin A, iron and zinc need to be developed, and assessed for their acceptability among target children.

**Objective:**

Homemade provitamin A carotenoids (PVACs), iron and zinc-rich complementary food (CF), common bean pumpkin blend (BPB) formulated from pumpkin (*Sweet cream*) and common bean (*Obwelu*) and PVAC-rich pumpkin blend (PB) from *Sweet cream* were prepared by expert peer mothers. This study compared child acceptability of BPB and PB (control).

**Methods:**

The crossover acceptability study randomly assigned Ugandan children 6 to 24 months old to either receive 100 g of BPB (*n* = 35) or 100 g of PB (*n* = 35) on day one. After a washout period of one day, children crossed over to receive either BPB (*n* = 35) or PB (n = 35). The amount of CF consumed, duration of consumption, and micronutrient intake were assessed. The CF was acceptable if children consumed ≥50 g (50%) of served food (100 g). A paired t-test was used to determine the mean differences within participants between BPB and PB. The level of statistical significant difference was set at a probability value of 5% (*p =* 0.05).

**Results:**

The mean consumption of BPB and PB was 53.9 g and 54.4 g, respectively. The mean duration for consumption of BPB and PB was 20.6 and 20.3 min, respectively. There was no significant difference in the amounts consumed, and duration of consumption in BPB and PB (*p* > 0.05). The mean intake of vitamin A was significantly higher (*p* < 0.00001) in PB (152.5 μgRAE) compared to BPB (100.9 μgRAE). The mean iron intake was significantly higher in BPB (1.1 mg) (*p* < 0.00001) compared to PB (0.3 mg). Furthermore, zinc intake was significantly higher (*p* < 0.00001) in BPB (0.58 mg) compared to PB (0.13 mg).

**Conclusion:**

A homemade complementary food, BPB, made from locally available common bean and pumpkin is rich in PVACs, iron and zinc and is acceptable to children in the age range of complementary feeding in Uganda.

**Trial registration:**

Pan African Clinical Trials Registry www.pactr.org as PACTR202002576768667.

Retrospectively registered.

Date of registration: 29/January/2020.

## Background

The burden of hidden hunger (micronutrient deficiencies) resulting from inadequate intakes of key micronutrients, particularly iron, zinc, and vitamin A, contributes to reductions in linear growth, vulnerability to infection, reduced cognitive function, and significant child morbidity and mortality in the developing world [1]. In developing countries, vulnerability to vitamin A deficiency iron deficiency and zinc deficiency begins during the period of complementary feeding, when children are fed complementary foods (CFs) deficient in vitamin A, iron and zinc [2, 3].

The period of complementary feeding begins at 6 to 24 months or beyond, when nutritious homemade foods are supposed to be given alongside breast milk to meet the increased nutritional demands for a child’s growth and development [4]. During the age range of complementary feeding, nutritional demands for vitamin A, iron and zinc increase [5–7], indicating that vitamin A, iron and zinc-rich CFs are necessary at this critical period of child growth and development [4]. However, in Uganda children are fed CFs, predominantly prepared from staple cereals and tubers such as white maize, cassava, sweet potatoes and yams [8]. Such staples are deficient in vitamin A, iron and zinc, and their consumption has been linked to deficiencies in vitamin A, iron and zinc [3]. A most recent study conducted in central rural Uganda, analysed the micronutrient content of CFs, and established that they were deficient in iron and vitamin A [9].

The World Health Organization (WHO) recommends that in order to combat micronutrient deficiencies such as vitamin A, iron and zinc deficiencies child caregivers should feed their children CFs formulated from animal source foods, fortified foods and food supplements [4]. This is plausible because animal source foods, fortified foods and food supplements are rich sources of iron, zinc and vitamin A [10–14]. However, the rural poor including child caregivers in Ugandan are unable to physically access or afford animal source foods, fortified foods and food supplements [15, 16]. Moreover, despite a high child vitamin A supplementation (VAS) coverage of over 65% in Uganda [17], the prevalence of vitamin A deficiency among Ugandan children less than five years old has unacceptably increased from 20.4% [18] to 32.6% [17] in the last decade. To this end, it is necessary to identify locally available and affordable food ingredients rich in vitamin A, iron and zinc and use them to prepare CFs rich in vitamin A, iron and zinc to supplement on nutrition specific interventions such supplementation and fortification. Therefore, this study selected common bean, *Obwelu* and pumpkin, *Sweet cream*, locally cultivated in rural Uganda [19, 20], for use in the home preparation of a novel provitamin A carotenoids (PVACs), iron and zinc rich complementary food (CF). The former was selected because common bean is a rich source of iron and zinc [21–24], whilst the latter is a rich source of PVACs [25–27]. PVACs, are an inactive form of vitamin A predominantly found in plant food sources [28]. However, when PVACs-rich foods such as pumpkin are consumed, the PVACs are bioconverted into retinol, the active form of vitamin A used by the body [29].

Testing the acceptability of novel CFs is necessary because it is a measure that can inform whether caregivers will feed the CF to their children and whether children will accept to ingest the CF [30]. However, several studies have used child caregivers to test the acceptability of CFs [31–34], hence neglecting the target group for CFs, which are children 6 to 24 months old. Such studies argue that they prefer using caregivers to children because children are too young to provide a rational judgement on the sensory attributes such as taste, aroma, colour and texture, usually used to test for acceptability of foods [34]. However, caregiver acceptability of a CF does not guarantee child acceptability [35]. It is worth noting that child acceptability can be assessed by feeding the novel CF to the child, followed by measuring the amount of CF consumed, and the duration taken to complete the CF [36–39]. A novel PVACs, iron and zinc-rich homemade CF, common bean pumpkin blend (BPB) prepared from locally available common bean and pumpkin in Uganda is acceptable to child caregivers [40]. However, child acceptability of BPB is unknown. Children 6 to 24 months old are the target consumers for CFs [41]. Therefore, this study assessed the acceptability of a novel multiple micronutrient rich homemade complementary food, BPB among Ugandan children in the age range of complementary feeding (6 to 24 months old).

## Methods

### Study setting

This study was conducted in rural Kyankwanzi district, central Uganda [42]. The total population of Kyankwanzi district is 214,693, of which 34% are illiterate, 48% are females, 16% are child mothers 12–19 years old and 19% are children 0–4 years old [42]. Children in this study area are fed CFs deficient in vitamin A, iron and zinc [8, 9]. The preparation of the CFs and child acceptability study were conducted at Ntwetwe Health Centre IV, Kyankwanzi district.

### Description of the intervention

#### Ingredients for preparation of the complementary foods used in the intervention

This study formulated two homemade CFs, common bean pumpkin blend (BPB) and pumpkin blend (PB). The former was formulated from cooked common bean (*Obwelu*) and pumpkin (*Sweet cream*), whilst the latter (control CF) from cooked pumpkin, *Sweet cream*. The PB was selected as a control because pumpkin is commonly used as a single CF in Uganda [43].

**Fig. 1:**
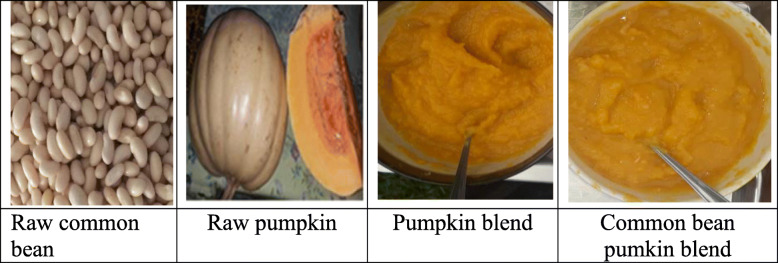
Study complementary foods and their ingredients used to prepare them

These ingredients were chosen because common bean is rich in iron and zinc [21–24], and pumpkin is rich in PVACs [25–27]. Moreover, these ingredients are cultivated in rural Uganda and available in the local markets [19, 20]. Figure 1 shows BPB, PB and ingredients, common bean (*Obwelu*) and pumpkin (*Sweet cream*) used to prepare BPB and PB.

#### Preparation of BPB and PB

At household level, caregivers usually prepare homemade CFs based on consistency (thinness and thickness) of the food and the child’s age and development as recommended by the 2017 Food and Agriculture Organization of the United Nations (FAO) guide to conducting participatory cooking demonstrations to improve complementary feeding practices [44, 45]. The 2017 FAO guide to conducting participatory cooking demonstrations to improve complementary feeding practices, encourages participatory cooking demonstrations involving community nutrition and health workers, mother-leaders and peer counsellors [44]. To this end, BPB and PB were prepared by child caregivers (expert peer mothers). Community health workers identified 10 expert peer mothers from the local community and invited them to Ntwetwe Health Centre IV to participate in the preparation of CFs used in the acceptability study. Expert peer mothers were encouraged to prepare CFs using the locally acceptable home-based methods used in the community to prepare common bean and pumpkin for child consumption.

Common bean (*Obwelu*) and pumpkin (*Sweet cream*) were purchased from the local market with assistance from expert peer mothers. Expert peer mothers prepared *Sweet cream* by peeling and discarding seeds followed by boiling the pulp. For *Obwelu*, expert peer mothers used overnight soaking (for about 8 h), followed by boiling (for about 1.5 h). After cooking, expert mothers indicated that they prepare homemade CFs in their community based on consistency (thinness or thickness of food) suitable for the child’s stage of development. To this end, after cooking by expert peers mothers, research assistants mixed the ingredients to form CFs based on the consistency as suggested by caregivers and recommended guidelines for conducting participatory cooking demonstrations to improve complementary feeding practices [44]. Research assistants prepared three different varieties of BPB by mixing and mashing *Sweet cream* and *Obwelu* together. Table 1 shows the ratio of mixing *Sweet cream* and *Obwelu* that was used to prepare BPB varieties.

**Table 1:** Ratio of mixing *Sweet cream* and *Obwelu* to formulate BPB

BPB varieties	BPB −1	BPB-2	BPB-3
*Sweet cream*: *Obwelu*	1:1	1:2	2:1

*BPB* Common bean pumpkin blend

After preparing, the three varieties of BPB were put on a table in three different serving dishes and presented to expert peer mothers. Based on consistency, expert mothers, one by one entered the room and where asked to choose one variety of BPB they would choose to feed their children, 6 to 24 months old [44]. All the 10 expert mothers unanimously selected BPB-3, prepared by mixing 2 parts of *Sweet cream* and 1 part of *Obwelu*. Mashed cooked pumpkin in Uganda is usually given as a single CF [43]. Therefore, pumpkin blend (PB) as a control was prepared from *Sweet cream*. Triplicate samples of prepared BPB (test food) and PB (control) were transported to METLAB East Africa limited laboratory, Kampala, Uganda for PVACs, iron and zinc analysis.

#### Vitamin A, iron and zinc analysis of BPB and PB

The PVACs content was analysed by high performance liquid chromatography (HPLC) as described in the HarvestPlus hand book for carotenoid analysis [46]. To analyse the vitamin A content, the Institute of Medicine (2001) bioconversion rates of PVACs to vitamin A, retinol (retinol activity equivalents) were used, i.e. 12 μg of β-carotene is equivalent to 1 μg of retinol, whilst 24 μg of α-carotene is equivalent to 1 μg retinol [47]. Iron and zinc concentrations of CFs were determined by flame atomic absorption spectroscopy (FAAS) as described elsewhere [9, 48]. Triplicate analysis for BPB and PB were done separately to get the mean content of PVACs, iron and zinc in each of the two CFs.

#### Micronutrient composition of BPB and PB

The mean concentrations of PVACs, iron and zinc were calculated per 100 g of edible portion of CF. Table 2 shows the PVACs, iron, zinc and vitamin A content per 100 g of edible portion of BPB and PB.

**Table 2:** Micronutrient composition of edible portion of BPB and PB

Micronutrient	BPB/100 g	PB/100 g
Iron (mg)	1.99	0.57
Zinc (mg)	1.08	0.23
β-carotene (μg)	2219	3326.5
α-carotene (μg)	50.5	75.1
Vitamin A, μg RAE	187	280.3

*BPB* Common Bean Pumpkin Blend; PB: Pumpkin Blend

RAE is Retinol activity equivalent (retinol)

RAE = β-carotene (μg/100 g)/12+ α-carotene (μg/100 g)/24 [47]

#### Study participants, enrolment, inclusion, and exclusion criteria

All children (aged 6 to 24 months old) coming for growth monitoring and immunisation at Ntwetwe Health Centre IV, Kyankwanzi district Uganda were screened for nutritional status and presence of any illness. Upon fulfilling the enrolment criteria (age 6 to 24 months, on complementary feeding) and obtaining consent for participation from the caregivers, the children were randomly allocated to two different study groups (BPB and PB) and children were enrolled. Children did not meet the enrolment criteria if their weight for age or weight for height z-score was < − 3 standard deviations, if they had any childhood acute illness or features suggestive of any chronic disease such as tuberculosis, any congenital anomalies such as cleft lip or palate.

#### Sample size determination

A maximum 50 g of CF per serving is considered adequate for children in the age range of complementary feeding [41, 49]. Therefore, the sample size was determined to test the hypothesis that the mean consumption of CF during the acceptability test would be at least 50 g (50%) of the amount offered (100 g). Assuming a mean difference of 5 g between test CF and control, and a standard deviation (SD) of 10 g in a normally distributed population of children 6 to 24 months old, a sample size of 63 for each CF would therefore allow us to reject the null hypothesis with 80% power. However, this was a crossover study meaning that participants consumed both test CF and control. In order to cater for loss to follow-up, an additional seven participants were added to the 63 to make 70 participants. To this end, the same 70 participants were enrolled in each group of BPB and PB.

#### Study design

**Fig. 2:**
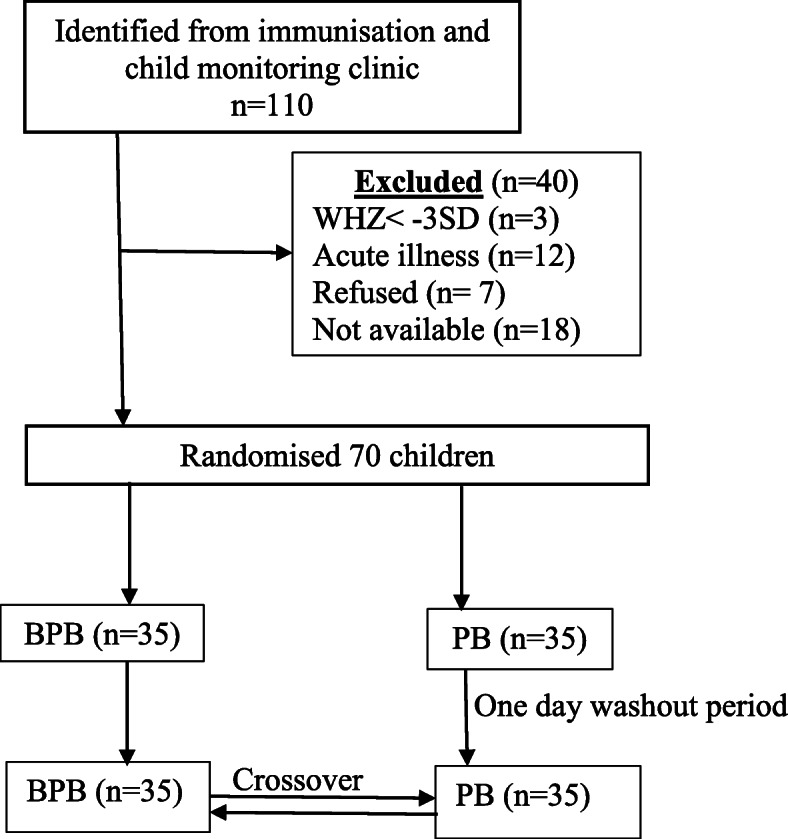
Child acceptability study design

This was a randomised crossover acceptability study**.** A total of 110 children from the growth monitoring and immunisation clinic at Ntwetwe Health Centre IV, Kyankwanzi district Uganda were identified for randomisation (Fig. 2). Out of the 110 children, 70 were eligible and assigned to BPB (intervention) and PB (control) using simple random sampling according to computer-generated random numbers. Computer-generated numbers were given to participants by a research assistant who was located off site. On the first day, 35 children were assigned to each group of BPB and PB. A wash out period of one day was granted, and on the third day participants crossed over to the opposite CF group. Figure 2 shows the study design.

#### Measurement of study outcomes

Child acceptability was assessed by feeding the novel CF to the child, followed by measuring the amount of CF consumed, and duration taken to complete the CF [36–39]. The primary outcome of the study was to measure the amount of CF consumed by children. The two secondary outcomes were to measure the time taken by the child to consume the served CF; and to analyse the PVACs, iron and zinc intake of each child based on the amount of CF consumed. Caregivers were also requested to report any discomfort or adverse effects experienced by the children after being fed the study CFs.

#### Amount of complementary food consumed by children

This study ensured that children were offered the assigned CF (BPB or PB) at least 1 h after they were last fed. A portion of 100 g of CF was offered to the child in a serving dish by the caregiver. The CF was considered acceptable if the child ingested at least 50 g of offered food [41]. The amount of food ingested was calculated by subtracting the left-over from the offered amount. Pre-weighed napkins were provided; any food that was regurgitated, vomited or spilled was swabbed, the napkin weighed and subtracted from the weight of the amount offered.

#### Duration of feeding

Duration of feeding was measured as described elsewhere [36]. Caregivers were asked to spoon feed their children the assigned CF until the child refused to eat. After a two-minute pause, the same food was offered a second time until s/he refused again. After a second two-minute pause, the food was offered a third time until refused again. After this third refusal, the feeding episode was considered terminated. The duration of feeding (excluding the intervening ‘pause periods’) was recorded by stopwatch, and the total duration of the feeding was noted. The feeding episode took place under the direct supervision of a trained research assistant to ensure that feeding was not forced. Children were considered as refusing intake if they moved their head away from the food, cried, clamped the mouth shut or clenched the teeth, or became agitated, spat out the food or refused to swallow as done elsewhere [36].

#### Micronutrient intake measurement

The micronutrient intake (MNI) for each child was calculated using the formula, MNI = A(g)*B/100, where A was the amount of CF (BPB or PB) consumed by the child and B was the nutrient composition in 100 g of CF food served to the child (see Table 2). For example, if the child consumed 50 g of 100 g of BPB served, then MNI for iron, zinc and vitamin A would be 50*1.99/100 (0.995 mg), 50*1.08/100 (0.54 mg) and 50*187/100 (93.5 μgRAE), respectively.

#### Measurement of background characteristics

Data on background characteristics such as age, gender and nutritional status of study participants were collected. Age was calculated in months based on the difference between the date of visit and date of birth. If exact date of birth of the child was unknown, the month and year of birth were estimated using a local events calendar. In such cases, age was calculated after imputing the day of birth as the 15th of the month, as recommended by 2019 WHO guidelines [50]. Date of birth was extracted from the child’s immunisation and growth monitoring chart. Nutritional status was determined using anthropometry, and diagnosed by Z scores based on the 2019 WHO recommendations for data collection, analysis and reporting on anthropometric indicators in children under 5 years old [50]. A child was stunted, wasted and underweight if his or her length for age Z score (LAZ), weight for length Z score (WLZ) and weight for age Z score (WAZ) was below − 2 standard deviations of the WHO reference respectively [51].

### Data analysis

Statistical and data analysis was done by STATA version 13.1. Background characteristics of the participants were evaluated by using descriptive statistics. The mean ± SD of the amount of the CF consumed, duration of consumption, and MNI was calculated. The paired t-test was used to detect the mean differences of outcome variables within participants between BPB and PB. The level of significant difference was set at a probability value of 5% (*p* 0.05).

### Ethical approval

Permission to conduct the study was granted by the District Health Office, Kyankwanzi district, Uganda. In South Africa, ethical approval was obtained from the Biomedical Research Ethical Committee, University of KwaZulu-Natal, South Africa (Reference number: BE 438/19). In Uganda, ethical approval was granted by The AIDS Support Organisation Research Ethical Committee (Reference number: TASO-REC/066/19-UG-REC-009). This randomised control trial was registered by Pan African Clinical Trials Registry (www.pactr.org) as PACTR202002576768667. Informed and signed consent were obtained individually from caregivers of child participants in the study, and all data were coded to remove identifying information and ensure confidentiality.

## Results

### Description of study participants

On day one, 35 children were either fed on BPB or PB, day two was a wash out period. On day three, 35 participants crossed to receive either BPB or PB. A total of 70 eligible children were enrolled and completed the acceptability test. They included 37 girls (52.9%) and 33 boys (47.1%), and their mean age ± SD was 12.3 ± 3.9 months. The proportion of wasting, underweight and stunting among study participants was 7, 17 and 29.3%, respectively. Caregivers did not observe any discomforts or adverse effects from their children after tasting BPB or PB. The mean age and SD of child caregivers was 23.6 years and 6.1, respectively. Of the 70 caregivers, 63 (90%) and 7 (10%) caregivers were females and males, respectively. Table 3 shows the socio-demographic characteristics and nutritional status of child participants.

**Table 3:** Socio-demographic characteristics and nutritional status of the acceptability study child participants

Characteristics	Participants (*n* = 70)
Socio-demographic	
Age (months), mean ± SD	12.3 ± 3.9
Gender	
Female, *n* (%)	38 (54.3)
Male, *n* (%)	32 (45.7)
Nutritional status	
Wasted	
Yes, *n* (%)	5 (7.1)
No, *n* (%)	65 (92.9)
Underweight	
Yes, *n* (%)	8 (11.4)
No, *n* (%)	62 (88.6)
Stunting	
Yes *n* (%)	27 (38.6)
No *n* (%)	43 (61.4)

*SD* Standard deviation

### Acceptability test

The aim of this study was to assess the acceptability of a novel homemade complementary food (BPB), rich in PVACs, iron and zinc, compared to PVAC-rich PB (control). Acceptability was measured by the amount of CF consumed by the child and duration of consumption. Table 4 shows results from the child acceptability test.

**Table 4:** Child acceptability and micronutrient intake between BPB and PB

Variable	BPB (*n* = 70)	PB (*n* = 70)	*p* value
Amount consumed, g (mean ± SD)	53.9 ± 2.97	54.4 ± 3.51	0.44
Feeding duration, minutes (mean ± SD)	20.6 ± 1.4	20.3 ± 1.6	0.14
Iron received in consumed food, mg (mean ± SD)	1.1 ± 0.59	0.3 ± 0.02	< 0.00001
Vitamin A received in consumed food, μg RAE (mean ± SD)	100.9 ± 0.7	152.5 ± 1.2	< 0.00001
Zinc received in consumed food, mg (mean ± SD)	0.58 ± 0.04	0.13 ± 0.01	< 0.00001

*BPB* Common Bean Pumpkin Blend, *PB* Pumpkin Blend, *SD* Standard Deviation

*μgRAE* Microgram Retinol Activity Equivalent (Retinol, Vitamin A)

### Amount of complementary food consumed

Children consumed on average 54.2 ± 3.3 g of served food. The mean consumption of BPB and PB was 53.9 g and 54.4 g, respectively. There was no significant difference in the amount consumed between the multiple micronutrient test CF and control CF (*p* = 0.44). The CF was acceptable if the child ate 50 g (50% and above) of the 100 g of CF offered. To this end, both BPB and PB were 100% acceptable to the study children.

### Duration of consumption of BPB and PB

The mean duration for consumption of BPB was slightly longer (20.6 min) compared to PB (20.3 min). However, there was no significant difference in mean duration of consumption for BPB and PB (*p* = 0.14).

### Vitamin A, iron and zinc intake from consumed BPB and PB

The mean vitamin A (retinol) intake was 100.9 μgRAE and 152.5 μgRAE in BPB and PB, respectively. The mean intake of vitamin A was significantly higher in PB compared to BPB (*p* < 0.00001). The mean iron intake was significantly (*p* < 0.00001) higher in BPB (1.1 mg) compared to PB (0.3 mg). Furthermore, zinc intake was significantly higher in BPB (0.58 mg), compared to PB (0.13 mg).

## Discussion

Expert peer mothers formulated a multiple micronutrient CF, BPB, rich in PVACs, iron and zinc, based on locally available, culturally acceptable food ingredients, that is, pumpkin and common bean based on the guide to conducting participatory cooking demonstrations to improve complementary feeding practices [44]. Provitamin A carotenoids are an inactive form of vitamin A [29, 47]. To this end, this study included the vitamin A content of CFs based on the 2001 Institute of Medicine bioconversion rates of PVACs to retinol, an active form of vitamin A used by the body [47]. Common bean pumpkin blend was superior in iron and zinc compared to PB because of the common bean mixed with pumpkin to form BPB (see Table 2). This is plausible because common bean is a rich source of iron and zinc [21–24]. Besides, PB was superior in vitamin A because 100% of PB was prepared from pumpkin, a rich source of PVACs [25–27].

The average recommended amount of CF per serving for a child, 6 to 24 months old during complementary feeding is 50 g [49]. The hypothesis was that the CFs would be acceptable if children consumed 50 g or more of the offered CF. The mean amount consumed of both CFs was above 50 g, indicating that both CFs were 100% acceptable. Moreover, there was no significant difference in the mean amount consumed between the BPB and PB.

Preparation and acceptability of micronutrient-rich CFs from locally available food ingredients has been reported previously [32, 33, 51, 52]. Based on the amount consumed, Bauserman and colleagues established that micronutrient-rich CF prepared from caterpillar, corn and palm oil was acceptable to children in the age range of complementary feeding, in the Democratic Republic of Congo [51]. Bauserman and colleagues developed the CF in accordance with the international standards on the formulation of foods intended for infants and children up to 2 years of age as outlined in Codex Alimentarius [41, 51]. However, this present study developed CFs based on consistency as determined by child caregivers and as recommended in the preparation of homemade CFs [44]. Preparation in accordance with the international standards on the formulation of foods intended for infants and children up to 2 years of age outlined in the Codex Alimentarius is more objective to nutrient content of CFs, compared to the subjective method of using consistency. This may explain why the iron and zinc content in the study by Bauserman and colleagues is higher than that in BPB [51]. The vitamin A (in the form of retinol activity equivalent), iron and zinc content of BPB was reported in this study. However, Bauserman and colleagues only reported zinc and iron content of their CF, despite the use of PVAC-rich palm oil as one of the ingredients in the formulation of the CF [28, 51]. Therefore, it is difficult to conclude the vitamin A content in the CF developed by Bauserman and colleagues [51].

In Kenya, the highest proportion of children aged 6 to 24 months old consumed over 75% of the CF developed from locally available termites and small fish, which was regarded as acceptable [52]. However, the Kenyan study also reported the estimated content of iron and zinc, but not vitamin A. In South Africa, CFs, were developed from provitamin A-biofortified foods [32, 33]. However, these studies did not test the acceptability of these CFs in the target age group (children 6 to 24 months old) of complementary feeding [32, 33]. In contrast to other previous studies [32, 33, 51, 52], this study developed a multiple micronutrient CF rich in vitamin A, iron and zinc, and tested its acceptability among children in age of complementary feeding. It is worth noting that vitamin A, iron and zinc are the leading three micronutrients of public health importance in the developing world needed to prevent child morbidity and the common childhood morbidities such as diarrhoea, respiratory tract infections, night blindness, and iron deficiency anaemia [1]. To this end, formulating and testing child acceptability of a PVACs, iron and zinc rich BPB was necessary in the developing country, Uganda.

The role of CFs in meeting the dietary reference intakes such as the recommended dietary allowance (RDA) for children in the age range of complementary feeding is well recognised [53]. The RDA is the intake that meets the nutrient need of almost all (97 to 98%) individuals in a group [47]. The RDA for retinol (vitamin A), iron and zinc for a child 13 to 24 months old is 300 μgRAE/day, 7 mg/day and 3 mg/day, respectively [47]. This study showed that the average intake of vitamin A, iron and zinc in one serving from BPB was 109.5 mg, 1.1 mg and 0.58 mg, respectively. This suggests that one serving of BPB would contribute 37, 16 and 19% towards meeting the RDA for vitamin A, iron and zinc, respectively in children 13 to 24 months old. It is worth noting that 100 g of cooked common bean used to prepare BPB contains 7.1 mg and 2.7 mg of iron and zinc, respectively [54]. This suggests that pumpkin mixed with common bean to formulate the BPB variety selected by mothers reduced iron and zinc content of common bean by 5 mg and 1.7 mg, respectively.

If BPB was served to children twice daily, it would contribute 74, 32 and 38% towards meeting the RDA for vitamin A, iron and zinc, respectively in children under study. It is worth noting that provitamin A-biofortified food crops such as maize and orange-fleshed sweet potato are bred to provide 50% of the mean daily vitamin A dietary requirement through normal consumption habits [55, 56]. However, BPB that is served twice daily would provide over 50% of the mean daily vitamin A RDA for a child, 13 to 24 months old. Iron and zinc concentration of BPB was low compared to other studies that developed and tested child acceptability of iron and zinc-rich complementary foods [51, 52]. However, there is convincing evidence from a systematic review study that low dose daily iron and zinc intake has a positive effect on iron and zinc status of children, 6 to 24 months old [57].

It is worth noting that the rural poor in Uganda feed their children CFs such as staple cereals and tubers, which are deficient in vitamin A [8, 9], because they lack physical and economic access to vitamin A-rich foods such as animal source foods and vitamin A-fortified foods [15]. To contribute towards combating vitamin A deficiency among in Ugandan children, bi-annual (6 monthly) high dose (200,000 IU or 60,000 μgRAE) VAS programmes have been running for more than a decade [17, 58, 59]. However, the prevalence of child vitamin A deficiency in Uganda has increased from 20.4% [18] to 32.6% [17] in the last decade. This is plausible because standalone VAS programmes may not combat vitamin A deficiency [60], because the liver is unable to store the high dose of 60,000 μg retinol (200 times the RDA for a child 12 to 24 months old), and therefore, the excess vitamin A is destroyed by the liver and excreted [61]. Moreover, the rise in serum retinol resulting from 6-monthly VAS is small, short-lived, and lasts only for 1 to 3 months [62]. To this end, a PVACs, iron and zinc rich BPB or a PVAC-rich PB may be necessary to complement VAS programmes in the fight against child vitamin A deficiency in Uganda.

### Strengths and limitations of the study

Bioavailability is defined as the proportion of the ingested nutrients that are absorbed in the small intestine, enter the circulation, and become available for utilization or storage in organs [63]. This study did not analyse for the anti-nutrient compounds such as phytic acid in BPB. It is worth noting that common bean, an ingredient of BPB is a potential source of phytic acid, an anti-nutrient that reduces the bioavailability of iron and zinc [2, 64]. However, common home cooking methods such as soaking and boiling used in this study reduce the amounts of phytic acid in common bean [65]. Moreover, zinc absorption is not associated with dietary phytic acid intake in infants and young children in the age range of complementary feeding [66]. Furthermore, PVACs are fat soluble [63], and therefore, incorporating fat during preparation of BPB or PB could have improved their PVACs bioavailability. However, the widely accepted 2001 Institute of Medicine bioconversion recommendations of PVACs to retinol used in this this study are independent of the use of fat as an ingredient in the preparation of PVACs-rich foods [47]. Furthermore, this present study considered that the offered CF was acceptable to children, 6 to 24 months old, if they ingested at least 50 g of the CF offered. This is in line with the 2013 FAO and WHO guidelines on formulated CFs for older infants and young children, which considers 50 g of offered CF as a reasonable maximum quantity which children in the age range of complementary feeding can ingest per feeding [41]. However, child acceptability findings from the present study with regard to the amount of CF ingested in a specified duration should be interpreted with caution. This is because a wide range of 10 g to 50 g of a formulated CF is considered a reasonable quantity an older infant or a young child in the complementary feeding stage can ingest easily in one feeding session for a specified duration, depending on age [41]. Therefore, older infants (6 to 12 months old) may ingest less CF in a specified duration than young children (13 to 24 months old).

## Conclusion

Expert peer mothers (child caregivers) developed a PVACs, iron and zinc-rich complementary food, BPB based on locally available pumpkin and common bean by using home preparation methods. The newly developed, multiple micronutrient rich BPB is acceptable to children in the age range of complementary feeding, and has the potential to contribute towards combating deficiencies in vitamin A, iron and zinc among children aged 6 to 24 months old.

## Data Availability

The datasets used and/or analysed during the current study are available from the corresponding author on reasonable request.

## Abbreviations

BPB: Common Bean Pumpkin Blend
CF: Complementary Food
CFs: Complementary Foods
MNI: Micronutrient Intake
PB: Pumpkin Blend
PVACs: Provitamin A Carotenoids
RAE: Retinol Activity Equivalent
RDA: Recommended Dietary Allowances
SD: Standard Deviation
VAS: Vitamin A Supplementation
